# Bend‐insensitive fiber optic ultrasonic tracking probe for cardiovascular interventions

**DOI:** 10.1002/mp.16334

**Published:** 2023-03-09

**Authors:** Sunish J. Mathews, Callum Little, Edward Zhang, Paul Beard, Tara Mastracci, Roby Rakhit, Adrien E. Desjardins

**Affiliations:** ^1^ Wellcome/EPSRC Centre for Interventional and Surgical Sciences University College London London UK; ^2^ Department of Medical Physics and Biomedical Engineering University College London London UK; ^3^ Department of Cardiology Imperial College Healthcare NHS Foundation Trust London UK; ^4^ Department of Cardiology Royal Free London NHS Foundation Trust London UK

**Keywords:** bend‐insensitive fiber, cardiovascular interventions, fiber optic ultrasound sensor, transesophageal electrocardiography, ultrasonic tracking

## Abstract

**Background:**

Transesophageal echocardiography (TEE) is widely used to guide medical device placement in minimally invasive cardiovascular procedures. However, visualization of the device tip with TEE can be challenging. Ultrasonic tracking, enabled by an integrated fiber optic ultrasound sensor (FOUS) that receives transmissions from the TEE probe, is very well suited to improving device localization in this context. The problem addressed in this study is that tight deflections of devices such as a steerable guide catheter can result in bending of the FOUS beyond its specifications and a corresponding loss of ultrasound sensitivity.

**Purpose:**

A bend‐insensitive FOUS was developed, and its utility with ultrasonic tracking of a steerable tip during TEE‐based image guidance was demonstrated.

**Methods:**

Fiberoptic ultrasound sensors were fabricated using both standard and bend insensitive single mode fibers and subjected to static bending at the distal end. The interference transfer function and ultrasound sensitivities were compared for both types of FOUS. The bend‐insensitive FOUS was integrated within a steerable guide catheter, which served as an exemplar device; the signal‐to‐noise ratio (SNR) of tracking signals from the catheter tip with a straight and a fully deflected distal end were measured in a cardiac ultrasound phantom for over 100 frames.

**Results:**

With tight bending at the distal end (bend radius < 10 mm), the standard FOUS experienced a complete loss of US sensitivity due to high attenuation in the fiber, whereas the bend‐insensitive FOUS had largely unchanged performance, with a SNR of 47.7 for straight fiber and a SNR of 36.8 at a bend radius of 3.0 mm. When integrated into the steerable guide catheter, the mean SNRs of the ultrasonic tracking signals recorded with the catheter in a cardiac phantom were similar for straight and fully deflected distal ends: 195 and 163.

**Conclusion:**

The FOUS fabricated from bend‐insensitive fiber overcomes the bend restrictions associated with the FOUS fabricated from standard single mode fiber, thereby enabling its use in ultrasonic tracking in a wide range of cardiovascular devices.

## 1 INTRODUCTION

In minimally invasive cardiovascular interventions, steerable guide catheters are commonly used for accessing a wide range of vascular and cardiac targets. These interventions include structural heart repair to treat diseased valves, and cardiac ablation to treat atrial fibrillation. Deflection at the distal end, actuated by a rotary collar at the proximal end, allows for maneuvering of the catheter and deployment of devices in specific directions. In this context, transesophageal echocardiography (TEE) is often used as an image guidance modality owing to its high contrast for soft tissues such as the heart and vascular tissue, and real‐time 2D and 3D imaging.^1^, ^2^, ^3^, ^4^ Identifying the location of the guide catheter tip relative to the TEE imaging probe can be crucial for successful outcomes. However, with TEE, this identification can be very challenging, as the tip can readily stray from 2D imaging planes and can have poor echogenicity.^5^ Even with 3D TEE, accurately identifying the distal ends of devices can be very challenging.^6^, ^7^

Ultrasonic tracking is a method for localizing an interventional device relative to an external imaging probe that has recently undergone rapid development. This method involves ultrasonic communication between the device and the ultrasound (US) imaging probe, in order to measure the time delays between transducer elements in the probe and an integrated transmitter/receiver in the device. Several recent innovations have been centered on needle‐based US‐guided interventions in fetal surgery and regional anesthesia,^8^, ^9^, ^10^, ^11^, ^12^, ^13^ including a system for dynamically adjusting the electronic focus of the US imaging probe to the needle tip.^14^ To date, there has been a paucity of studies exploring US tracking of devices during intracardiac and endovascular interventions.^6^

Fiber optic ultrasound sensors (FOUS) are generally well suited to ultrasonic tracking. Recently, FOUS comprising high finesse Fabry‐Pérot cavities with high sensitivities (Noise Equivalent Pressure < 100 Pa across 0.5 to 20 MHz), wide detection bandwidths (up to 40 MHz), and largely omnidirectional frequency responses were demonstrated.^15^, ^16^ Typically fabricated from standard single‐mode fiber, which comprises a core that transmits a single spatial mode of light, their advantages in minimally invasive applications include small lateral dimensions (outer diameter < 200 µm), low fabrication costs, and immunity to electromagnetic interference.^17^ However, a key challenge that arises with the use of a FOUS in cardiovascular applications is that they can experience bending that exceeds the fiber specifications, for instance during tight turns in vessels, or with device deflections to access specific intracardiac locations.

The use of bend insensitive (BI) fibers allows for sensing when integrated into highly deflected devices.^18^, ^19^ BI fibers were previously used to fabricate fiber optic hydrophone or ultrasonic sensors.^20^, ^21^, ^22^ However, in those sensors, the acoustic sensing element (coiled fiber) had an outer diameter of at least 10 mm, which is too large for minimally invasive cardiovascular interventions. Moreover, these sensors had low detection bandwidths (< 0.625 MHz) that is not well suited to diagnostic ultrasound imaging.

In this work, we demonstrated the feasibility of ultrasonic tracking in the context of TEE‐guided cardiac interventions by developing a FOUS fabricated with BI optical fiber. We assessed the static bending characteristics of this FOUS and of one based on standard single mode fiber. For validation, the FOUS fabricated with BI fiber was positioned within a steerable guide catheter and progressed into the left ventricle of a cardiac phantom.

## 2 METHODS

### 2.1 Overview of the catheter tip tracking using a transesophageal US probe and FOUS

**FIGURE 1:**
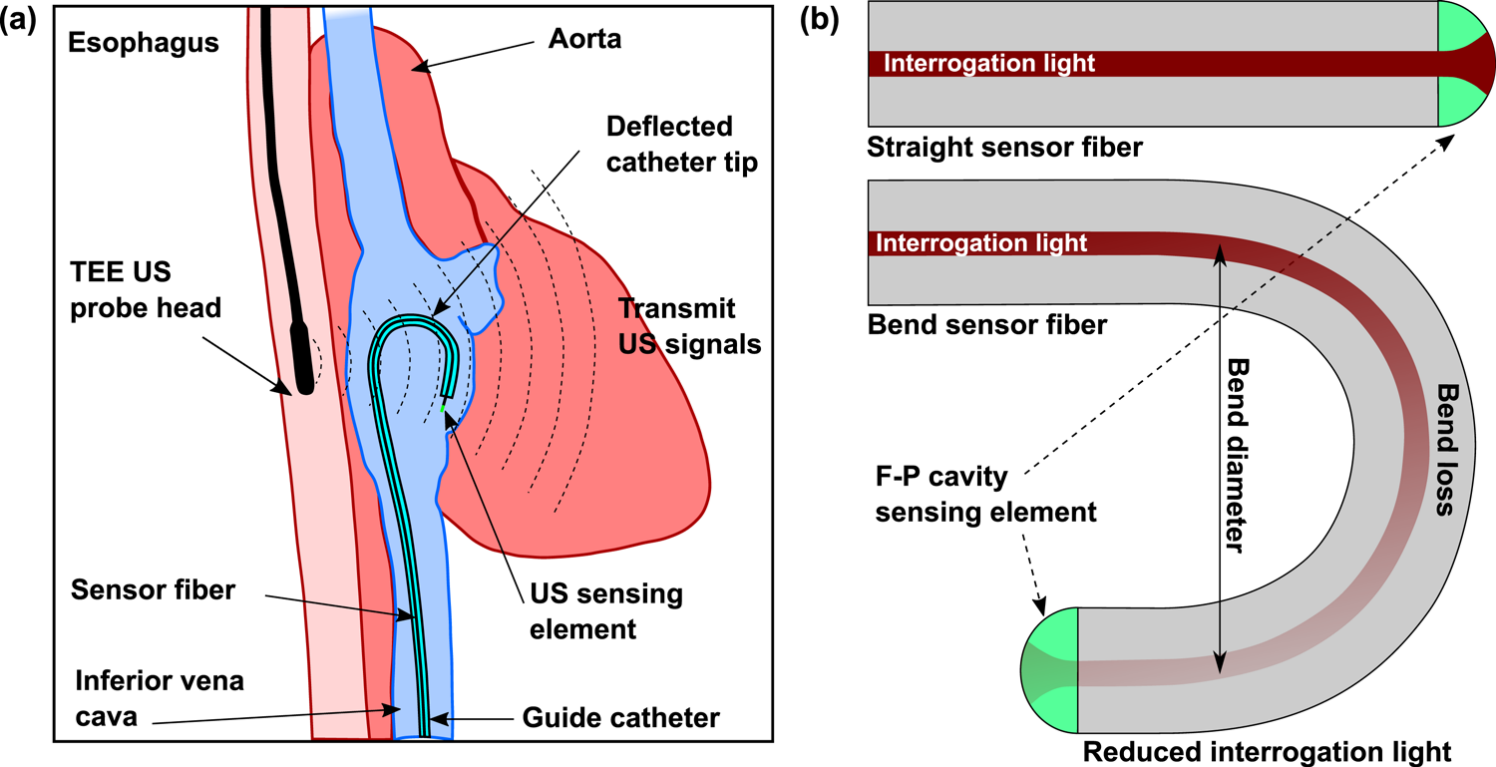
Schematic overview of the transesophegeal echocardiography (TEE) with US tracking of the steerable catheter (a), showing the steerable guide catheter positioned in the left ventricle, the TEE US probe within the esophagus adjacent to the heart and the catheter, and (b) the fiberoptic ultrasound sensor with a Fabry‐Pérot (F‐P) cavity‐based ultrasound sensing element at the distal end, in straight (top) and bent (bottom) configurations.

A schematic of a steerable tip guide catheter used in conjunction with TEE is shown in Figure 1a. A minimally invasive TEE probe is inserted into the oral cavity of the patient; its transducer head is positioned within the esophagus in close proximity to the heart. The FOUS integrated within the catheter receives US signals transmitted from this probe and the corresponding signals are used to estimate the coordinates of the catheter tip in the imaging plane of US probe. During deflection of the distal end of the catheter, the FOUS fiber undergoes bending, with resulting attenuation of the interrogation light (Figure 1b). The detection sensitivity of the FOUS is dependent on the Fabry‐Pérot (F‐P) interference transfer function (ITF), which is the relationship between reflected optical power and the phase. The sensitivity will scale linearly with incident optical power to the F‐P cavity.^23^

### 2.2 Fabrication of FOUS and intravascular tracking probe

Two types of FOUS were fabricated, using standard SMF with outer diameter (OD) ∼ 250 µm (SMF28e, Fiberstore, UK) for one type and BI SMF with OD ∼ 155 µm [SM1250B3(9.8/125)P, Fibercore, UK] for the other. The ultrasound sensing element of the FOUS was formed at the distal end of the fiber by depositing a multi‐layer thin film coating which comprises a polymer layer sandwiched between two reflective coatings to form a high‐finesse Fabry‐Pérot (F‐P) cavity, using methods previously described.^16^, ^23^ Sensing is performed via thickness modulation of the F‐P cavity with the incident US waves, which is detected as corresponding modulations of the intensity of the interrogation light reflected from the F‐P cavity.^24^

The FOUS fabricated from BI fiber was integrated into an ultrasonically transparent sheath with an inner diameter of 2 mm (K‐201, Olympus, UK), with the tip of the FOUS retracted ca. 150 µm from the tip of the sheath. The luer connector at the proximal end of the sheath was paired with a side arm adapter (Touhy‐Borst, Cook Medical, Ireland). The fiber was routed through the straight section of the adapter; deionized water was injected via the side port to flush the sheath, thereby ensuring US coupling to the FOUS. The sheath was inserted into the steerable tip catheter, with the sheath extending from distal end of the catheter by approximately 3 mm.

### 2.3 Interference transfer function with static bending

In static tests, the FOUS fiber was subjected to a 180° deflection at around 3 cm from the F‐P cavity, forming a U‐shape with one half‐turn. The bend radius was varied in steps by fixing one point of the fiber and linearly translating another point with a translation stage. The bend radius was reduced from 12.5 mm to 2.5 mm in increments of 2.5 mm and the ITF was measured at each bend radius. The interference transfer function (ITF) was measured using an interrogation system comprising an external cavity wavelength‐tunable laser and a custom‐built photo‐receiver system described by Zhang et al.^23^

The US sensitivity of the FOUS with bending at distal end was compared for both types of FOUS. Each FOUS fiber was wound once around optical post with a circular cross‐section and a diameter of 6 mm (Thorlabs, UK), to introduce a bend radius of 3 mm in a region of the fiber spanning 3 cm to 1.5 cm from the distal end (Figure S1a). The F‐P cavity of the FOUS was then positioned inside a water tank at 2.2 cm in front of a calibrated planar transducer (3.5 MHz, Precision Acoustics, UK). To maintain the measurement conditions for both types of FOUS, the optical sensitivities were matched, by adjusting the interrogation laser power at which the FOUS was operated. The SNRs of the detected US signals were compared for straight and fully deflected distal ends. The SNR valued were estimated as a ratio of the peak signal to the standard deviation of the noise floor (linear scale). To estimate the peak signal, 100 averages were performed.

### 2.4 Overview of the ultrasonic tracking system

The US tracking system comprised an US imaging system (Ultrasonix MDP, BK Medical, UK) with two probes: a phased array TEE probe (mTEE8‐3/5; center frequency at 6 MHz; 16 elements) and a side‐viewing linear array laparoscopic probe (LAP9‐4/38; frequency range 4–9 MHz; 128 elements). The laparoscopic probe was used as a surrogate to a clinical‐grade TEE probe^25^, ^26^ to obtain high‐quality cardiac US images. With eight times more transducer elements and a US lens for elevational focus, this laparoscopic probe produced far higher quality US images than the research‐grade TEE probe used in this study. This TEE probe was chosen for its compatibility with the research‐grade programmable system.

The US tracking system also comprised a custom‐built console that received reconstructed B‐mode images from the US imaging system. The console also acquired two trigger signals from the US imaging system (start of each B‐mode frame; start of each A‐line) concurrently with FOUS signals. The FOUS signals were recorded as a time‐series signal for each frame. The “time‐of‐flight” delays between transmission by the imaging probe (line trigger) and reception by the FOUS were mapped to the corresponding distance using the speed of sound (1500 m/s). The time series signals corresponding to transmissions from one image were concatenated to form a 2D tracking image and displayed in real‐time. The catheter tip positions were estimated from this image based on the centre of mass (CoM) of a region of interest (100 × 100 pixels) around the positions of the maxima. To obtain an accurate representation of the shape of the catheter tip image, background subtraction followed by thresholding such that pixels with less than ∼ 70% of the maximum intensity were zeroed. The CoM was then estimated based on the average of all pixels weighted by the intensity at each position in the region of interest.

### 2.5 Guide catheter tracking in a cardiac phantom

**FIGURE 2:**
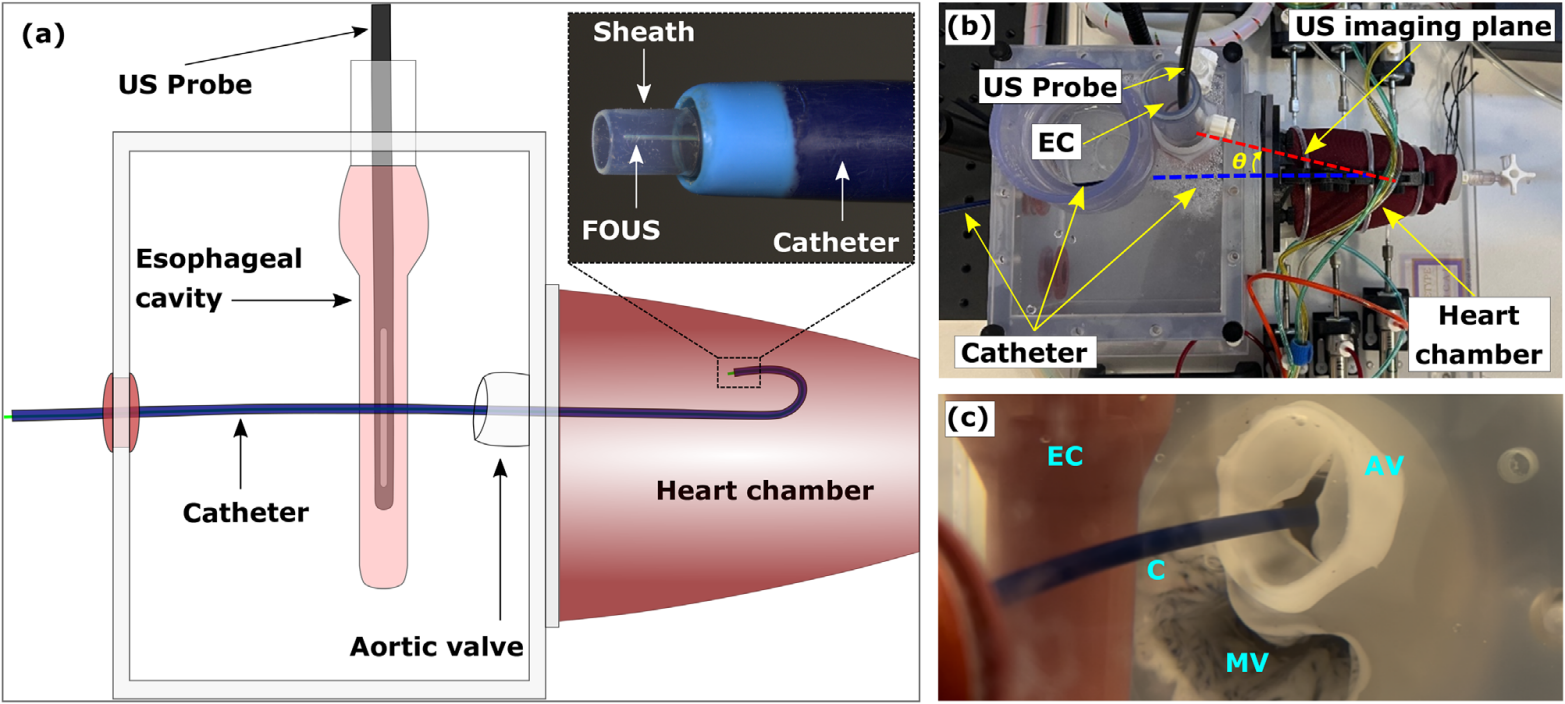
(a) Schematic of the heart valve phantom with the steerable tip catheter positioned in the left ventricle via the aorta, and the laparoscopic ultrasound (US) probe positioned in the esophageal cavity. The inset shows a microscope image of the distal end of the tracking probe, with the catheter (blue), the sheath (clear) and the distal end of the fiberoptic ultrasound sensor (FOUS). (b) A photograph showing the top‐view of the heart phantom and experimental setup, with the orientation of the US imaging plane (red‐dotted line) with respect to the catheter axis (blue‐dotted line) making an angle θ which was about 30°, and (c) A close‐up photograph of the heart chamber from the left side showing the catheter (C) progressed through the aortic valve (AV), with the mitral valve (MV) below and the esophageal cavity (EC) on the left.

The FOUS was positioned within a steerable guide catheter (TG0705509, Medtronic, UK) with an inner diameter of 7F (2.3 mm) and a usable length of 55 cm. A microscope image of the distal end of the catheter with the tracking probe is shown in Figure 2a. The guide catheter was positioned inside the left ventricle of a custom‐made dynamic heart valve phantom (Archetype Biomedical Inc., Canada) filled with deionized water (Figure 2a). It had side ports for inserting catheters into the left ventricle, and an esophageal cavity in which to position the US imaging probe.

The US imaging probes were positioned inside the esophageal cavity of the phantom. Figure 2b shows the photograph of the experimental setup from the top; Figure 2c shows a close‐up view of the heart chamber opening. The tracking signals were acquired with the catheter tip in straight and fully deflected position. For both case the electronic focus of the US probe was set to ∼ 5.5 cm to coincide with the depth of the catheter tip. In the fully deflected position, the catheter was bent to a bend radius of ∼ 4.5 mm. For both cases, the tracking signals were acquired over 100 US frames. The SNR for the tracking signals for each frame was estimated as a ratio of the peak signal to the standard deviation of the noise floor (linear scale). The latter was estimated from a manually‐ chosen region of the 2D tracking image without any apparent signal. The mean SNR was then calculated from the individual SNR values obtained from each of the 100 frames. In the case of the TEE probe, the US signals received from the FOUS were mapped onto a cartesian coordinate system based on the geometry of the TEE probe scan‐head and the field of view angle (90°).

## 3 RESULTS

### 3.1 Impact on ITF and ultrasound sensitivity with static bending

**FIGURE 3:**
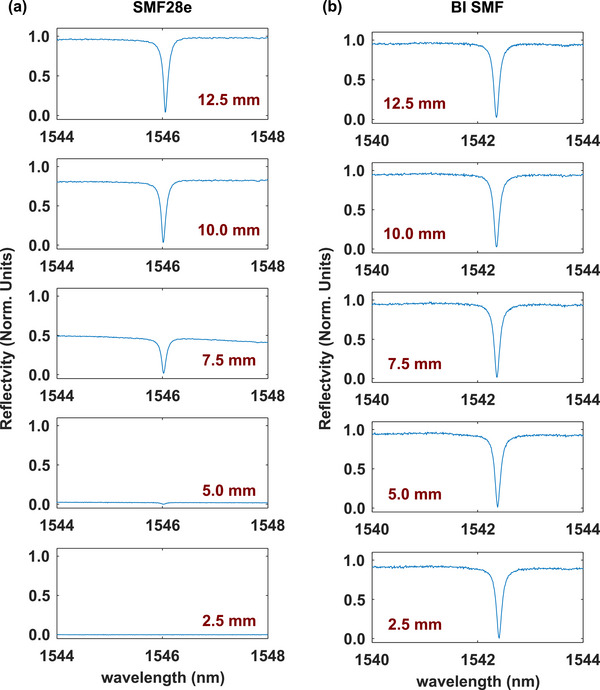
Interference transfer functions (ITFs) of the FOUSs [left side: SMF28e fiber; right side: bend‐insensitive (BI) fiber with static bending at their distal ends. The bending radii ranged from 12.5 to 2.5 mm (top to bottom).

The ITF plots measured for the FOUSs fabricated from standard SMF and BI fiber, obtained with bend radii ranging from 12.5  to 2.5 mm, are shown in Figure 3. For the FOUS based on standard SMF (Figure 3a), the normalized reflectivity dropped by 50% for bend radii below 10 mm. At a bend radius of 5.0 mm, less than 2% of the reflected power was received at the detector, relative to a straight fiber. For a bend radius at 2.5 mm, no reflected power was detected. By comparison, the FOUS fabricated from BI fiber measured a consistent ITF throughout this range of bend radii (Figure 3b). At maximal bending, with a bend radius of 2.5 mm, the reflectivity was 95% compared with a straight fiber.

The plots for the US signals detected by the FOUS are shown in the supplementary materials figures (S1b‐S1d). The FOUS based on standard SMF yielded an SNR of 45.8 when the distal end was straight. By comparison, with a bend radius of 3 mm at the distal end, no signal from reflected light was detected, due to the high bend‐induced light loss. The FOUS fabricated from BI fiber yielded an SNR of 47.7 with a straight distal end; the SNR was 36.8 when the bend radius was 3 mm.

### 3.2 US catheter tip tracking in the heart valve phantom

**FIGURE 4:**
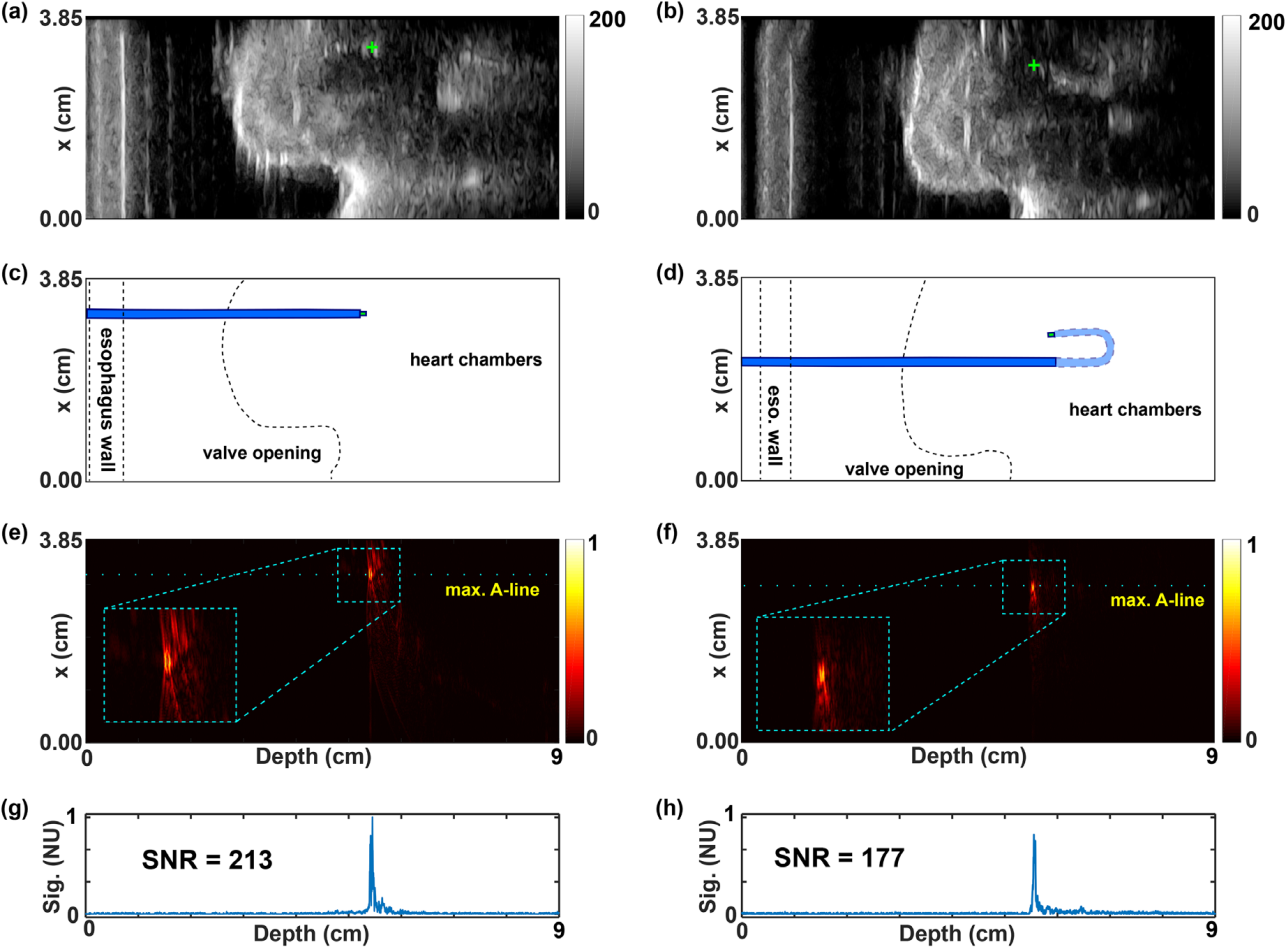
Catheter tip tracking in a heart valve phantom. Ultrasound images for a steerable guide catheter with straight (a) and fully deflected (b) distal ends, with the CoM‐estimated coordinates of the catheter tip ([3.26 cm, 5.44 cm] for the straight catheter and [2.92 cm, 5.57 cm] for the deflected catheter) automatically superimposed on the US images for both cases using green ‘+’ markers. Schematic showing the features of the phantom as observed in the US images, with the overlay of the catheter for straight (c) and fully deflected (d) distal ends. The corresponding 2D tracking images of the catheter tip formed by FOUS signals are shown for straight (c) and fully deflected (d) distal ends, with zoomed‐in views (insets). In each case, the frame with maximum intensity is shown here. The A‐lines with maximum intensities and SNR values, normalized to unity for the straight case, are plotted for straight (e) and fully deflected (f) ends. NU: Normalized Units.

On ultrasound imaging, the catheter tip, the interface of the esophageal cavity wall, and the valve of the phantom were apparent. The catheter tip image and the tracking signals are shown in Figure 4, for both straight and bent configurations of the guide catheter. The US images recorded for the straight and bend position of the catheter are shown in Figures 4a and 4b. The features on the US images together with the overlaid position of the catheter is shown in Figures 4c and 4d. The US imaging plane was oriented at ∼ 30° with respect to the catheter axis. The parts of the catheter that were above the US imaging plane are shown in solid and the parts that were below are shown in transparent.

The corresponding 2D needle tip images of the catheter formed by the signals received by FOUS are shown in Figures 4e and 4f for the straight and fully deflected distal ends, respectively. The A‐ line (time series data post envelope detection) along the maximum intensity are showed in Figures 4g and 4h. The SNR estimated for the case with straight distal end was 213; for the bend distal end it was 177. Across all 100 measured frames the mean SNR values were 195 (straight) and 164 (fully deflected) with a maximum variation of 10%. Despite poor image quality from the TEE probe (Figures S2a, S2b), high SNRs were obtained from the FOUS signals (Figures S2c, S2d): 31 for the straight distal end case and 23 for the fully deflected distal end. The better SNR estimated with the laparoscopic probe is due to the relatively high US power emitted by the transducer, which interfere constructively at the position of the catheter tip due to the US focus set at same depth.

## 4 DISCUSSION

The bend insensitivity of the fiber used in this study is conferred by a low refractive index trench around the cladding of the fiber to reflect the leaked mode back to the core of the fiber as it undergoes bending. The ITF recorded for the FOUS based on BI fiber at various bend radii suggests that the shape of the ITF doesn't undergo appreciable change with bending. As per the International Telecommunications Union—Telecommunications (ITU‐T) Sector standard G.657.B3, the minimum bend loss of 0.2 dB per turn is specified at a bend radius of 5 mm. However, this study showed that the FOUS remained operational down to a bend radius of 2.5 mm. By comparison, the commercial steerable guide catheter used in this study, which was chosen to be highly deflectable relative to other cardiovascular devices, has a minimum curve radius of 4.5 mm (achieved with a 180° deflection at the distal end).

The insensitivity of the ITF to fiber bending suggests that the ultrasonic sensitivity and directivity of the F‐P cavity are also bend insensitive. Indeed, the acoustic properties of the F‐P cavity sensor are defined by the interactions of the US field with the F‐P sensing element (< 50 µm in thickness) and the first few 100 µm of the backing fiber immediately behind the sensor.^27^ As a result, the tracking resolution is not likely to be affected by fiber bending proximal to the F‐P sensing element. Indeed, the lateral component of the tracking resolution is primarily determined by the lateral confinement of the transmitted US field, whereas the axial component is determined by the frequency bandwidth of the FOUS, as previously demonstrated.^14^ For the TEE probe used in this study, the lateral resolution was lower than that of the laparoscopic probe due to the smaller imaging aperture, which gave rise to significantly less lateral confinement of the ultrasound beam.

In this study, fiber bending was not induced at the distal end; it occurred at distances more than 1 cm from the F‐P cavity. However, during a minimally invasive cardiovascular procedure, device deflection could change the orientation of the sensor relative to the imaging probe. While the BI FOUS is largely omnidirectional (−165° to 165°) over the diagnostic frequency range of 0.5–20 MHz (Figure S3), small deviations from omnidirectionality could result in changes in the tracking axial resolution: as catheter is deflected relative to the external imaging probe, there could be corresponding changes to the angle at which impinging ultrasound waves are incident on the distal end of the optical fiber.

The cardiac phantom used in this study was developed with synthetic materials for TEE visualization of the mitral valve and left ventricle.^28^ The experimental study did not include motion of the vascular tissue and there was absence of blood mimicking fluid which could have provided more realistic US attenuation. One limitation of the phantom was the absence of anatomical structures encountered prior to the mitral valve and left ventricle, such as the inferior vena cava (Figure 1).

In an in vivo setting, the FOUS may detect artefactual signals that arise from reflections of the transmitted US beams from tissues and devices within the body. Depending on their shapes and magnitudes, these artifacts may be removed with the use of CoM‐based estimation of the coordinates of the catheter tip. In the presence of highly echogenic structures, reflection artefacts could potentially be removed by image processing methods, such as those implemented with photoacoustic imaging of medical devices.^29^, ^30^

With 2‐D tracking with a linear array imaging probe as implemented in this study, the use of a single FOUS is not sufficient to provide the shape or direction of the catheter and there is insufficient information to localize it outside of the imaging plane. However, 3‐D ultrasonic tracking is likely to be an important step for clinical translation. This could be affected with side elements adjacent to a linear array, as demonstrated by Xia et al.^12^ Beyond 3‐D tracking, the use of more than one FOUS could provide tracking of multiple locations of a microcatheter, thereby yielding information about the device orientation relative to the imaging probe.

## 5 CONCLUSION

In this study, we demonstrated the viability of receive‐mode ultrasonic tracking with a FOUS in the context of TEE‐guided intracardiac procedures for the first time. This method of determining the device tip relative to the ultrasound probe could be particularly beneficial for structural heart interventions. A key enabling innovation in this study was the development of a bend‐insensitive FOUS that remained operational below bend radius ∼ 3 mm, which allows for its use with highly flexible intravascular devices that undergo tight bending during the surgical procedure. The use of FOUS in this context is significant because of the advantages they confer relative to electronic counterparts, such as device miniaturization, cost‐effectiveness, immunity to electromagnetic interference, compatibility with magnetic resonance imaging (MRI) and multiplexing capabilities. The small dimensions of the BI fiber used in this study (OD: 155 µm) make the FOUS compatible with a wide range of cardiovascular devices for ultrasonic tracking and optical ultrasound imaging.

The FOUS fabricated from bend‐insensitive fiber overcomes bend restrictions associated with the FOUS fabricated from standard single mode fiber, thereby enabling its use in ultrasonic tracking in a wide range of cardiovascular devices including catheters and guidewires. Beyond ultrasonic tracking, fiber optic ultrasound sensors with BI fiber could have broad relevance to other sensors and imaging devices for use in the body, for instance those used for optical ultrasound^31^ and photoacoustic imaging probes.^32^, ^33^

## CONFLICT OF INTEREST STATEMENT

A. E. D is a shareholder of Echopoint Medical Ltd.

## Supporting information


**FIGURE S1** US signals detected by FOUSs based on standard SMF and BI fiber, with SNR estimates based on windowing to isolate the first peak (14 – 15 µs), (a) Experimental setup used for measurements showing the US transducer head, the distal end of the FOUS in front of it, and the bend introduced in the FOUS fiber by winding it once over a 6 mm optical post (measurements were done with the setup immersed in a water tank), (b) signal detected by standard SMF based FOUS with straight distal end, (c) signal detected by BI fiber‐based FOUS with straight distal end, and (d) signal detected by the BI fiber‐based FOUS with bent distal end with bend radius ∼ 3.0 mm.

**FIGURE S2** Catheter tracking in heart valve phantom with US imaging using the mTEE probe, US image of the phantom with (a) straight catheter distal end, (b) bend distal end.

**FIGURE S3** Frequency dependent directivity map of the BI FOUS for incidence angles from −165° to 165°.
