# Supplementary figures and images for: Bend‐insensitive fiber optic ultrasonic tracking probe for cardiovascular interventions
Source: Med Phys. 2023 Mar 9;50(6):3490–7. doi: 10.1002/mp.16334 (PMC10615325; doi:10.1002/mp.16334)

(a)

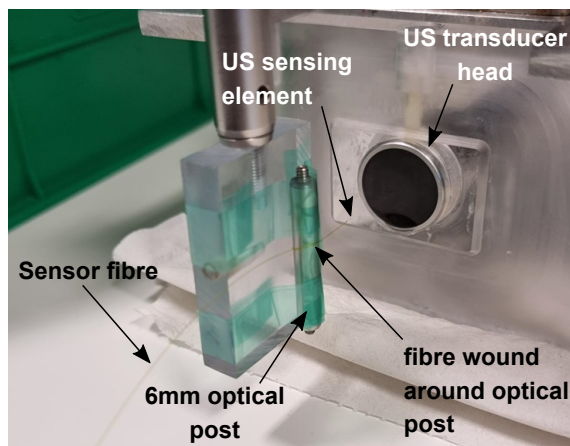

(b)

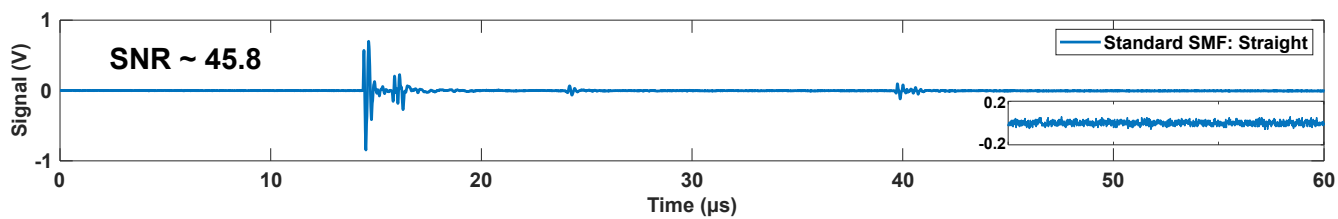

(c)

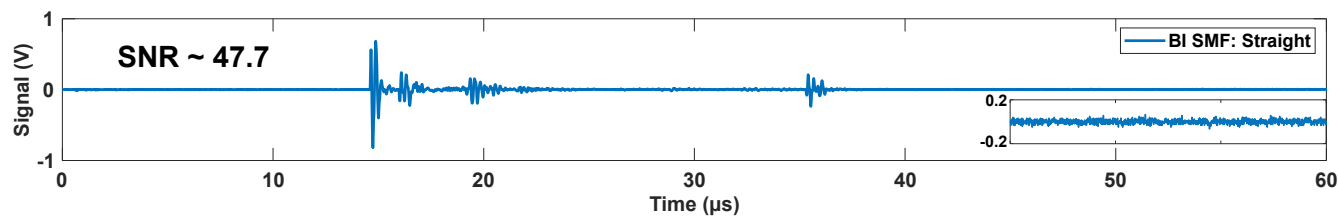

(d)

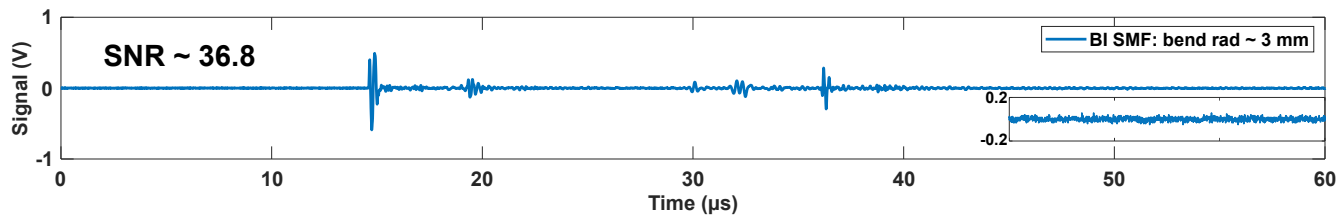

Supplement: Supplementary file 1 — FIGURE S1 US signals detected by FOUSs based on standard SMF and BI fiber, with SNR estimates based on windowing to isolate the first peak (14 – 15 µs), (a) Experimental setup used for measurements showing the US transducer head, the distal end of the FOUS in front of it, and the bend introduced in the FOUS fiber by winding it once over a 6 mm optical post (measurements were done with the setup immersed in a water tank), (b) signal detected by standard SMF based FOUS with straight distal end, (c) signal detected by BI fiber‐based FOUS with straight distal end, and (d) signal detected by the BI fiber‐based FOUS with bent distal end with bend radius ∼ 3.0 mm. [file MP-50-3490-s002.pdf]

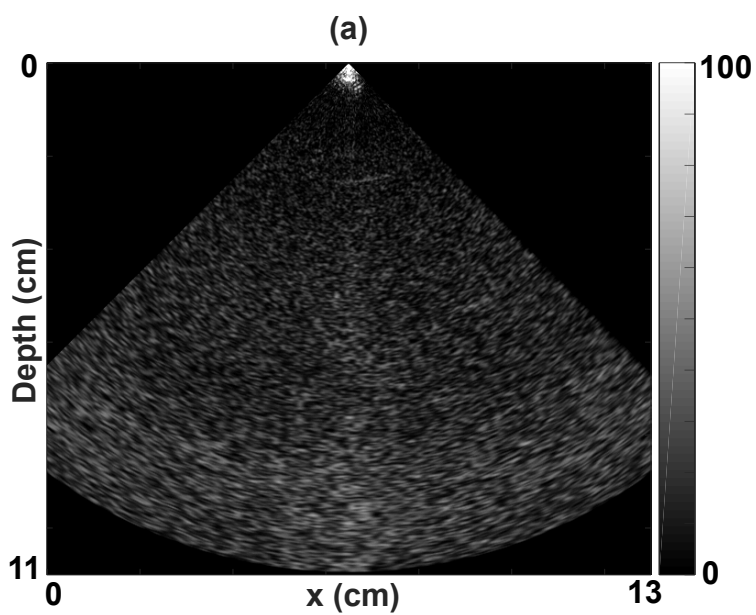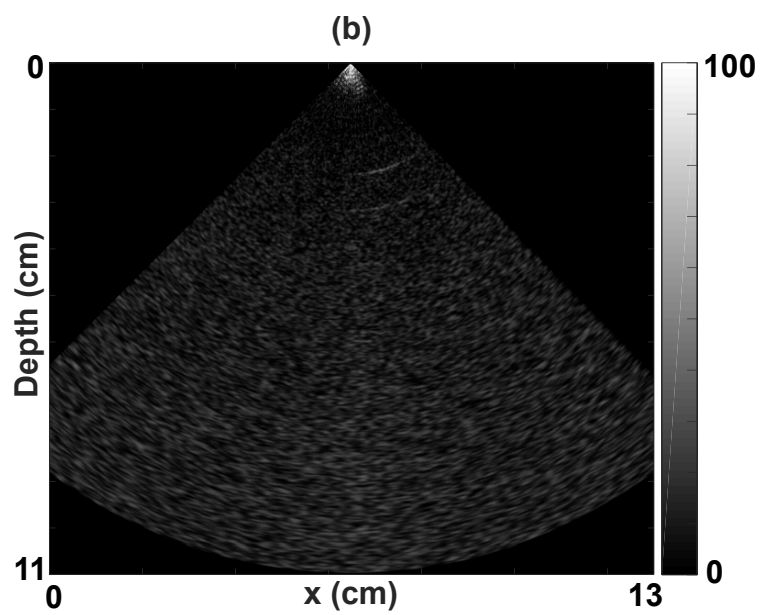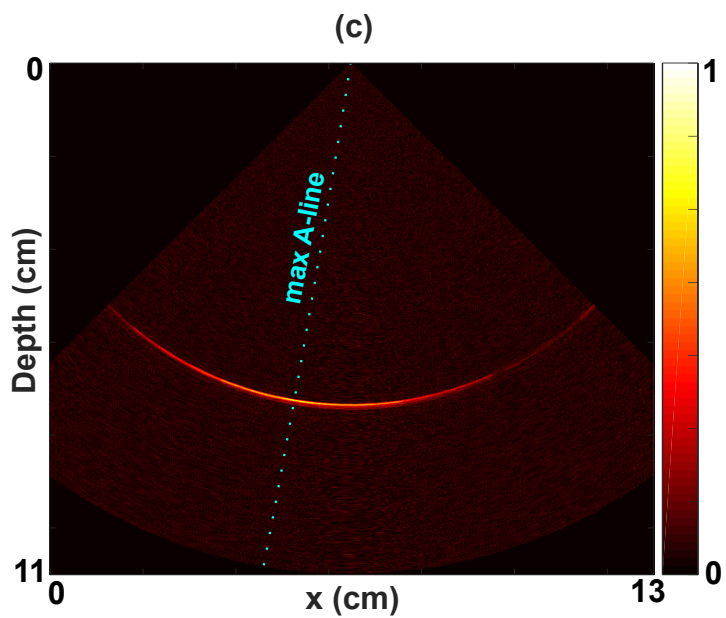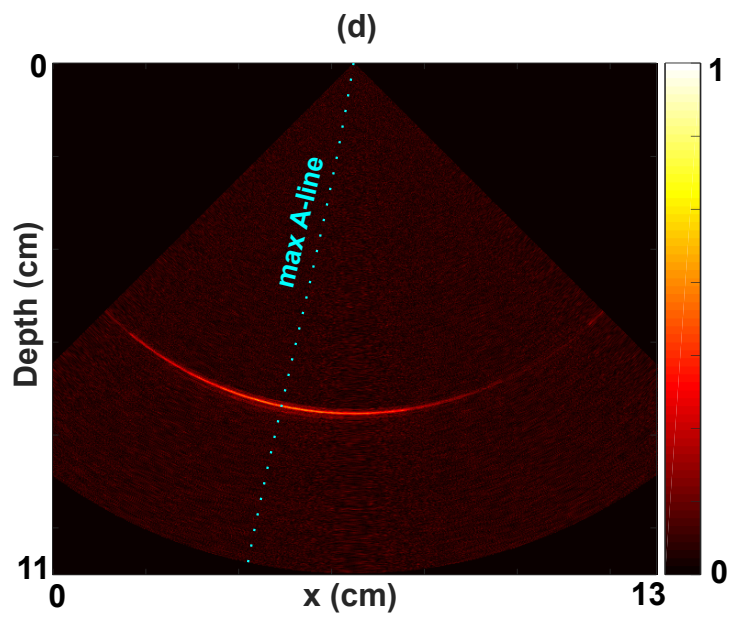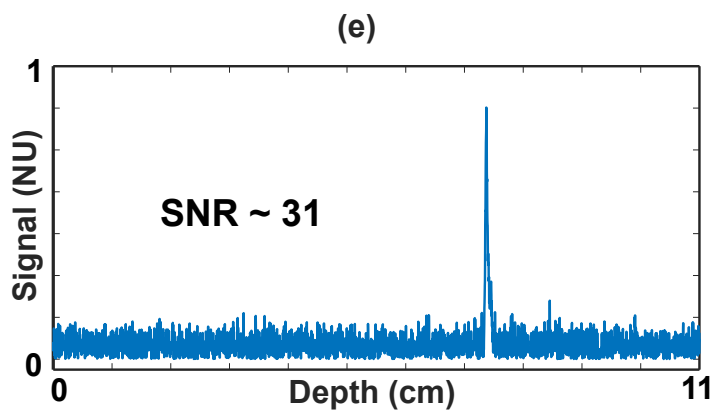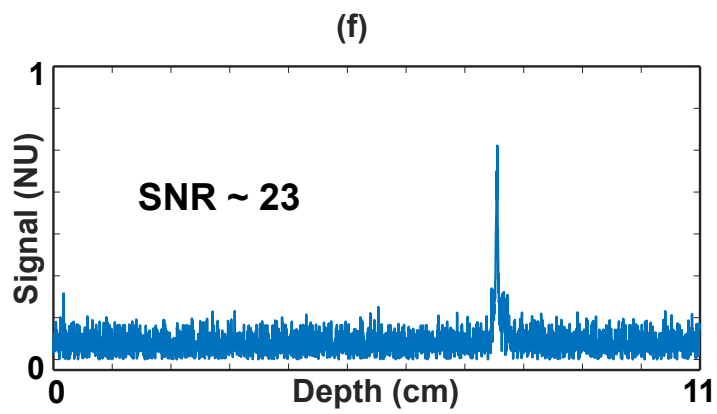

Supplement: Supplementary file 2 — FIGURE S2 Catheter tracking in heart valve phantom with US imaging using the mTEE probe, US image of the phantom with (a) straight catheter distal end, (b) bend distal end. [file MP-50-3490-s001.pdf]

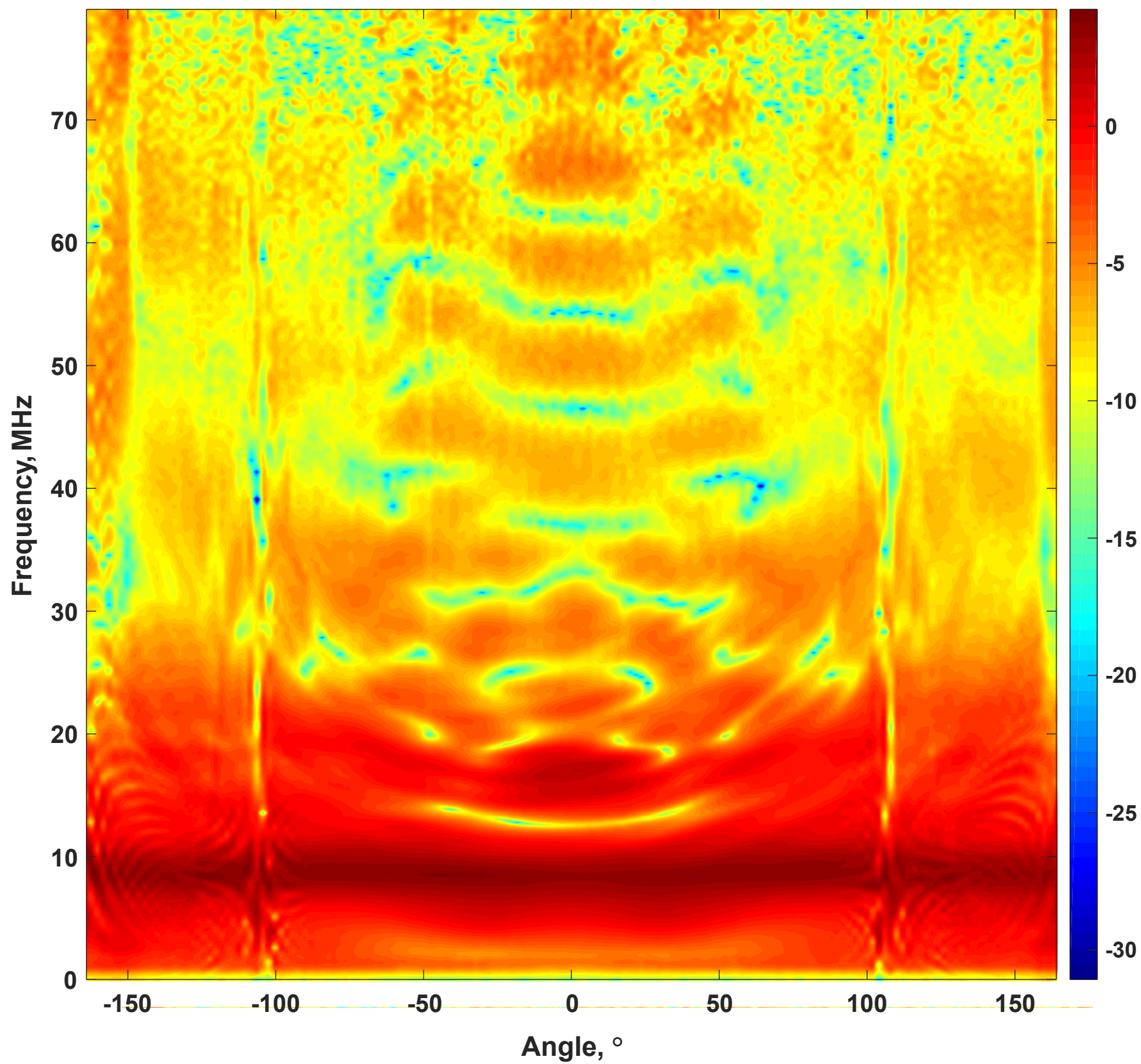

Supplement: Supplementary file 3 — FIGURE S3 Frequency dependent directivity map of the BI FOUS for incidence angles from −165° to 165°. [file MP-50-3490-s003.pdf]
